# Alpha-Synuclein Pathology and the Role of the Microbiota in Parkinson’s Disease

**DOI:** 10.3389/fnins.2019.00369

**Published:** 2019-04-24

**Authors:** Emily Fitzgerald, Sarah Murphy, Holly A. Martinson

**Affiliations:** ^1^WWAMI School of Medical Education, University of Alaska Anchorage, Anchorage, AK, United States; ^2^School of Medicine, University of Washington, Seattle, WA, United States

**Keywords:** Parkinson’s disease, microbiota-gut-brain axis, microbiota, alpha-synuclein, gut dysbiosis, innate immunity, blood-brain barrier, biomarkers

## Abstract

There is a principle in science, known as Occam’s razor, that says the correct solution is usually the one with the simplest explanation. The microbiota-gut-brain axis, an interdependent series of communication loops between the enteric nervous system (ENS), the microbiota, the gut, and the brain, offers important insight into how changes in our gut affect distant organs like our brains. The inherent complexity of this axis with the crosstalk between the immune system, inflammatory states, and the thousands of bacteria, viral, and fungal species that together make up the microbiota make studying the interactions that govern this axis difficult and far from parsimonious. It is becoming increasingly clear that the microbiota is integral to this axis. Disruption of the healthy flora, a phenomenon collectively referred to as dysbiosis, has been implicated as a driver for several diseases such as irritable bowel syndrome, rheumatoid arthritis, obesity, diabetes, liver disease, and neurological disorders such as depression, anxiety, and Parkinson’s disease (PD). Teasing apart these complex interactions as they pertain to PD is critical for our understanding of this debilitating disease, but more importantly, for the development of future treatments. So far, treatments have been unable to stop this neurodegenerative disease, succeeding only in briefly dampening symptoms and buying patients time before the inevitable loss of function ensues. Given that the 10 years prognosis for death or life-limiting disability with someone diagnosed with PD is upwards of 80%, there is a desperate need for curative treatments that go beyond symptom management. If PD does begin in the periphery with bidirectional communication between the microbiota and the immune system, as recent literature suggests, there is an exciting possibility that progression could be stopped before it reaches the brain. This systematic review assesses the current literature surrounding the role of the microbiota in the pathogenesis of alpha-synucleinopathies and explores the hypothesis that alpha-synuclein folding is modulated by the microbiota. Furthermore, we discuss how changes in the gut environment can lead to pathology and outline the implications that advances in understanding the interactions between host and microbiota will have on future research and the development of potential biomarkers.

## Introduction

Neurological disorders have become the leading cause of disability in the world, and as the fasting growing among them, Parkinson’s disease (PD) is one of the most significant medical and social burdens of our time (Dorsey and Bloem, 2018). With the aging population, the number of people suffering from PD is expected to double from 6.9 million in 2015 to 14.2 million in 2040 (Dorsey and Bloem, 2018). PD is a complex, progressive, and widely systemic neurodegenerative disease characterized by a number of motor and non-motor symptoms. Historically thought to be primarily a motor disorder, it is now evident that the disease is multifactorial, affecting many processes involved in endocrine signaling, autonomic control, memory, behavior, olfactory function, gastrointestinal motility, sleep, skin function, and pain perception (Braak et al., 2003a; Unger et al., 2016; Krüger et al., 2017). Michael Okun, MD, the Medical Director for the National Parkinson Foundation of the United States refers to PD as the “the most complex disease in clinical medicine.” The cardinal features of PD include bradykinesia, rigidity, postural instability, difficulty with gait, and a resting tremor that makes it difficult for patients to perform simple tasks such as tying a pair of shoes or typing on the computer. Non-motor symptoms of the disease include gastrointestinal dysfunction, cognitive dysfunction, autonomic dysregulation, olfactory dysfunction, fatigue, psychosis, sleep disturbances, dementia, and mood disorders (Bridi and Hirth, 2018; Caputi and Giron, 2018; Perez-Pardo et al., 2018a; Sun and Shen, 2018). While symptoms and progression of the disease vary with each patient, the vast majority of cases are insidious in onset. By the time patients begin noticing motor symptoms, the disease is usually advanced and a steady decline in function often follows. Recent studies suggest that non-motor symptoms, particularly those associated with gastrointestinal dysfunction may show up as much as 20 years before neurological changes appear. Indeed, the prevalence of this non-motor symptom phase is so high that it has been referred to as the prodromal phase of PD (Bridi and Hirth, 2018).

The defining pathology in PD is the destruction of dopamine producing neurons in a part of the midbrain called the substantia nigra. It is the loss of these dopamine producing neurons that is believed to drive the bradykinesia, tremor, and other motor symptoms associated with PD. The observation that proteins involved in synaptic transmission in the prefrontal, cingulate cortex and substantia nigra are altered in patients in the prodromal phase suggests that the non-motor symptoms are also caused by impaired synaptic transmission (Bridi and Hirth, 2018; Willard and Isett, 2019). Another hallmark finding of PD is an accumulation of neuronal inclusion bodies, known as Lewy bodies, in various parts of the brain and body (such as the substantia nigra, cerebral cortex, dorsal nucleus of the vagus nerve, sympathetic ganglia, and the myenteric plexus of the intestines). These Lewy bodies are round, dense, eosinophilic inclusion bodies made up of misfolded alpha-synuclein, ubiquitin, complement proteins, and cytoplasmic structural proteins. Under physiological conditions, alpha-synuclein exists as a soluble monomer and is thought to be involved in neurotransmitter release by interacting with vesicle fusion complexes to facilitate transport, release and reuptake of neurotransmitters (Calo et al., 2016). In contrast, the conformation of the alpha-synuclein protein in PD exists in a less soluble form that aggregates with other proteins. While the accumulation of misfolded alpha-synuclein is widely recognized as a central component of PD, the reason it accumulates is less clear and remains an active area of research.

Emerging studies on the subject of PD and the microbiota have begun to focus their attention on the potential mechanisms by which the microbiota contribute to the formation of alpha-synuclein pathogenic species in the enteric nervous system (ENS) and central nervous system (CNS). Functioning as the surveillance system for bacteria and viruses, pattern recognition receptors (PRRs), represent an important part of this work. With the knowledge that different metabolic products elicit focused responses in the host through activation of PRRs, a new theory in the pathogenesis of PD has emerged. Central to this theory is the idea that changes in the gut microbiota can lead to activation of these receptors and thereby contribute to disease. With the use of accessible gene mapping technology, it is now possible to compare the microbiota of healthy subjects to subjects with PD. Several clinical studies have found differences between abundance of certain gut microbiota in patients with PD compared to healthy subjects (Keshavarzian et al., 2015; Scheperjans et al., 2015; Unger et al., 2016). A recent study showed that fecal transplants from patients with PD into alpha-synuclein overexpressing mice significantly impaired motor function relative to alpha-synuclein overexpressing mice that had received fecal transplants from healthy subjects (Sampson et al., 2016). This observation not only suggests that the microbiota may be involved in the regulation of movement, but it also supports the hypothesis that alpha-synuclein pathogenic species may be dependent on the presence of certain types of bacteria.

Despite breakthroughs in our understanding of the complexity of the microbiota-gut-brain axis and the results of recent studies identifying changes in the gut microbiota of patients with PD, the conditions that lead to Parkinsonian pathology in humans are not well understood. In this review, the current research surrounding how misfolded alpha-synuclein contributes to Parkinsonian pathology will be reviewed at length. Then, potential mechanisms by which the microbiota influences the formation of alpha-synuclein will be examined with the aim of outlining conditions that lead to alpha-synuclein aggregation in the periphery.

## Alpha-Synuclein

Alpha-synuclein is a small protein made up of 140 amino acids. Originally discovered to be encoded by the SNCA gene in 1997, it was found that a single missense mutation in this gene gave rise to an autosomal-dominant form of PD (Polymeropoulos et al., 1997). While the most common form of PD is sporadic, mutations in SNCA and LRRK2 genes have been linked to autosomal-dominant forms of inheritance whereas mutations in Parkin, PINK1, DJ-1, and ATP13A2 genes have been associated with autosomal-recessive forms of inheritance (Lashuel et al., 2013; Bridi and Hirth, 2018; Larsen et al., 2018; Price et al., 2018). Studying individual mutations in SNCA and other genes has led to the identification of several molecular signaling pathways that are impaired in both genetic and sporadic forms of PD (Braak et al., 2018; Larsen et al., 2018).

The expression of alpha-synuclein appears to be developmentally regulated. For instance, during fetal development it is expressed in every organ, only later becoming concentrated in the nervous system and to a lesser extent blood cells and hematopoietic cells in adults (Ltic et al., 2004). The discovery that alpha-synuclein is abundant in the periphery during human development and continues to exist in the periphery into adulthood suggests that this protein may serve functions outside of the CNS. If alpha-synuclein is indeed involved in multiple peripheral pathways, this may explain the abundance of non-motor symptoms seen in virtually all patients with PD.

The physiology of alpha-synuclein is complex. In physiological states, it can be found in presynaptic terminals both in the cytoplasm of cells and directly bound to membranes (Calo et al., 2016).

There are three regions of the protein: an amphipathic N-terminal domain that interacts with phospholipid membranes, a hydrophobic central region and an acidic C-terminal region. The alpha-synuclein has been seen in monomeric, oligomeric and fibril conformations. These conformations have been well studied in the review: The many faces of alpha-synuclein: From structure and toxicity to therapeutic target by Lashuel et al. (2013). The fibril conformation has been identified as the pathogenic species in Lewy bodies but the role of the monomeric and oligomeric forms in the development of the fibril form is unclear. Since both the monomer and oligomer conformation have been seen in native conditions, it has been postulated that it is the disruption of the ratio of these forms rather than just the presence of the oligomer form alone that leads to aggregation and pathology (Lashuel et al., 2013; Miraglia et al., 2018). The multitude of conformations the protein adopts, depending on the surrounding environment, is in part what makes understanding its function difficult.

### Alpha-Synuclein: Function

Despite being widely present in the brain and to a lesser extent, other organs of the body, the idea that alpha-synuclein has an essential role in normal physiological functioning is relatively new. The presence of alpha-synuclein at synaptic membranes, an observation that has been confirmed through several immunohistochemical studies, has proven to be key in discovering the role alpha-synuclein has in neuronal communication (Maroteaux et al., 1988; Bellucci et al., 2008; Chu and Kordower, 2010; Peelaerts et al., 2015; Changyoun et al., 2016; Calo et al., 2016). Current knowledge suggests the protein is involved in maintaining homeostasis of neurotransmitter release by interacting with proteins involved in synaptic vesicle fusion and transportation (Calo et al., 2016). This is supported by the observation that changes in SNARE complexes, proteins that mediate vesicle transport, are seen in the brains of patients with alpha-synucleinopathies like PD (Bellucci et al., 2008; Calo et al., 2016). Aggregations of misfolded alpha-synuclein are thought to interfere with the formation of the SNARE complex, specifically resulting in problems with vesicle docking and fusion that ultimately lead to decreased neurotransmitter release and synaptic dysfunction (Calo et al., 2016). In a study by Holmqvist et al. (2014) transport of aggregated oligomeric forms of the protein rapidly increased following treatment with the microtubule destabilizing agent, vinblastine. This observation suggests that alpha-synuclein also interacts with microtubules. Additionally, other studies have shown that the activity of DAT, a dopamine transporter, decreases in the presence of aggregated alpha-synuclein (Bellucci et al., 2008; Bellucci et al., 2011; Bridi and Hirth, 2018).

In addition to playing a role in the transport of the neurotransmitter dopamine, alpha-synuclein appears to be critical for the development of dopaminergic neurons. In a study looking at the role of alpha-synuclein in the development of neurons, Garcia-Reitboeck et al. (2013) found that in the presence of endogenous mouse alpha-synuclein, wild-type mice had a higher number of dopaminergic neurons in the substantia nigra compared to mice with deletions of the alpha-synuclein gene. Consistent with this notion, two recent studies have found that the absence of normally functioning alpha-synuclein correlates to a decrease in the number of dopaminergic neurons observed in the substantia nigra (Garcia-Reitboeck et al., 2013; Calo et al., 2016). Collectively, these studies suggest that alpha-synuclein is critical for the regulation of dopamine and provide an explanation for why dopamine levels are decreased in the brains of patients with PD.

### Alpha-Synuclein: Aggregation and Pathology

Regardless of the ongoing debate about the exact form alpha-synuclein takes in the physiological state, it is known that the transition to the aggregated beta sheet conformation is a necessary step in Lewy body synthesis (Miraglia et al., 2018). It is unclear whether the pathology arises from the aggregated form of the protein being neurotoxic or as a result of the loss of normal function. The work of recent studies indicate that the alpha-synuclein pathogenic species spreads through the nervous system by inducing aggregation of alpha-synuclein in recipient cells in a prion-like manner that propagates from neuron to neuron (Ltic et al., 2004; Peelaerts et al., 2015; Changyoun et al., 2016; Dagher and Zeighami, 2018; Terada et al., 2018). Evidence supporting this theory was first observed in 2010 when post-mortem analyses of patient’s brains who had received fetal neuronal transplants as part of treatment revealed that the transplanted (and thereby presumed disease free) fetal tissue contained Lewy body pathology (Chu and Kordower, 2010). Even more striking, the degree of pathology observed was time dependent, with the older 14 years old grafts having the highest level of Lewy bodies and markedly fewer dopamine transporters (Chu and Kordower, 2010). Identifying the conditions that lead to aggregation of the neurotoxic forms of alpha-synuclein is therefore essential to understanding the pathology and progression of PD.

It should be noted that while there are a number of significant findings in support of retrograde transport of misfolded alpha-synuclein in a prion-like manner, the mechanisms underlying this propagation and subsequent seeding is an active area of research. For example, the presence of misfolded alpha-synuclein in the brain at one point in time does not mean that sustained pathology and progression into an alpha-synucleinopathy will necessarily follow. In an *in vivo* study following injection of pre-formed alpha-synuclein fibrils (PFFs) into the ENS of rats and non-human primates, alpha-synuclein pathology was observed in the brainstem of the rats at 1 month following injection, but neither the rats or non-human primates displayed the brainstem pathology at later periods of time (Manfredsson et al., 2018). This observation demonstrates that clearance of alpha-synuclein pathogenic species occurs in some cases, suggesting that disruption of this process may be contributing to PD and other alpha-synucleinopathies.

While the discussion concerning the spread of alpha-synuclein is ongoing, it is known that the presence of alpha-synuclein pathogenic species in the ENS is sufficient to induce colonic dysmotility in the gastrointestinal tract, and in some animal models, appears to positively correlate to motor impairment severity (Paumier et al., 2015; Manfredsson et al., 2018). For example, Manfredsson et al. (2018) found that direct injection of PFFs into the ENS of rats and non-human primates resulted in reduced colonic motility in the host. This observation is important because it demonstrates a causal relationship between alpha-synuclein pathology in the gut and gastrointestinal symptoms. These findings along with the high prevalence of gastrointestinal dysregulation reported in patients with PD suggest that alpha-synuclein pathogenic species may play a significant role in non-motor gastrointestinal symptoms.

### Alpha-Synuclein: Propagation

The idea that the gastrointestinal system is involved in PD is widely recognized and supported by clinical and empirical observations. The early involvement of the vagus nerve, the presence of constipation as an early symptom, the strong link between the gut and the dopamine producing reward system of the brain, and the fact that this reward system is one of the first parts of the brain to deteriorate as the disease progresses, are all consistent with the hypothesis that PD may originate in the gut (Perez-Pardo et al., 2017; Dagher and Zeighami, 2018). In 2003, Heiko Braak led a study looking at the brains of patients with autopsy confirmed PD. They observed that in addition to damage to specific subnuclei of the substantia nigra, a hallmark finding of PD pathology, damage was also always seen in the vagal nerves (Braak et al., 2003a). This was an important finding because prior to this, alpha-synuclein pathology was believed to be confined to the brain. Since then, the theory that PD may begin outside of the CNS (or at least exist co-manifest in the periphery) has gained popularity. In a recent study, Holmqvist et al. (2014) isolated three different types of alpha-synuclein (aggregated, monomeric and oligomeric) from the brain lysate of a patient with PD and directly injected them into the submucosa of the ENS in mice to test the hypothesis that alpha-synuclein could propagate from the ENS to the CNS by way of the vagus nerve. Not only did they observe that alpha-synuclein deposited in a retrograde fashion from the ENS to the vagus nerve and then to the brain, but it did so in a time dependent manner that varied depending on the form of alpha-synuclein injected (Holmqvist et al., 2014). Thereby providing evidence that retrogradely transported alpha-synuclein can, in fact, lead to the spread of alpha-synuclein from the periphery to the brain (Holmqvist et al., 2014). Importantly, this pattern of alpha-synuclein deposition mirrors the rostral to caudal pattern of destruction of dopaminergic neurons seen in the brains of patients with PD. These findings support Braak’s early proposal that PD could be the result of a pathogen such as a bacteria, virus or pathogenic proteins that has passed through the mucosal layer of the gastrointestinal tract, and gained entry into the CNS via unmyelinated preganglionic fibers of the vagus nerve (Braak et al., 2003b). The discovery that neurons in the ENS develop the same form of misfolded alpha-synuclein that accumulates in the CNS of patients with PD suggests that alpha-synuclein may be the pathogen Braak was looking for.

### Transport via the Vagus Nerve

The observation that the dorsal nucleus of the vagus nerve displays Lewy body pathology very early on in the course of PD has led to the emerging theory that the vagus nerve is involved in alpha-synuclein propagation. The vagus nerve exists as a pair of mixed (meaning they provide both motor and sensory innervation) cranial nerves composed mainly of parasympathetic fibers that are essential in controlling the parasympathetic component of the autonomic nervous system (ANS). In the context of the gastrointestinal system, the vagus nerve carries out many functions like providing neuromotor control of smooth muscles involved in peristalsis and facilitating secretion of enzymes and hormones involved in digestion. It also represents a critical link between the gut and brain by facilitating bidirectional communication between the ENS and the CNS. Direct introduction of human alpha-synuclein in the vagus nerve of mice using the adeno-associated virus (AAV) as a vector has been shown to cause alpha-synuclein expression in the neuronal bodies and neurites of the medulla oblongata (Ulusoy et al., 2013). Interestingly, there is evidence that patients who have undergone truncal vagotomies for the treatment of peptic ulcer disease appear to have a decreased risk of developing PD (Svensson et al., 2015).

### Alternative Modes of Transport

**FIGURE 1:**
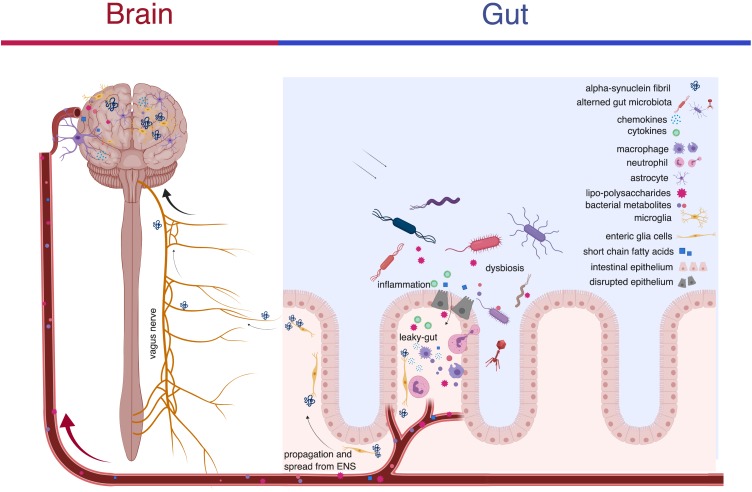
A schematic representation of the microbiota-gut-brain axis illustrating how alterations in the microenvironment of the gut contribute to changes in the CNS in PD. Changes in the microbiota may initiate a pathological process in the gut by (1) increasing the permeability of the blood-gut and blood-brain barriers thereby providing a route of transmission between the contents of the gut and the brain and (2) inducing formation of fibrillar alpha-synuclein pathogenic species. LPS and metabolites produced by the microbiota gain access to the CNS via the compromised barriers. The alpha-synuclein fibrils propagate to the brain via the vagus nerve where they induce other alpha-synuclein to undergo conformational change in a prion-like process. Alpha-synuclein and LPS also stimulate the release of pro-inflammatory factors such as: TNF-alpha, IL-lbeta, IL-2, IL-4, IL-6 from immune cells like macrophages, neutrophils and dendritic cells, increase expression of c-Jun, c-Fos, FosB, and cyclin B2 and promote production of chemokines like CCL2, CCL5, CCL17/TARC, CCL20, CXCL1, and CX3CL1 that are widely involved in neuroinflammation. These pro-inflammatory factors, chemokines and alpha-synuclein fibrils are powerful chemoattractants for microglia, neutrophils and astrocytes and may further impair normal ENS and CNS function. Created with BioRender.

Another proposed mechanism in the transport of alpha-synuclein pathogenic species to the brain is an endocrine like fashion. Support for this theory is grounded in the principle that bacteria in our gut produce short-chain-fatty-acids (SCFAs). These SCFAs directly affect the permeability of the blood-brain barrier and the blood-gut barrier (Manfredsson et al., 2018). The breakdown of these membranes and subsequent translocation of bacterial metabolites to the brain have been proposed as a mechanism driving neuroinflammation in the brain (Perez-Pardo et al., 2017; Figure 1). In a recent study investigating the relationship between SCFAs and PD, researchers administered bacterial derived SCFAs to alpha-synuclein overexpressing mice in an oral suspension to test the hypothesis that SCFAs drive the formation of alpha-synuclein in the brain (Sampson et al., 2016). Interestingly, researchers observed that the introduction of bacterial derived SCFAs induced significant motor deficits in the mice and generated alpha-synuclein reactive microglia in the brain (Sampson et al., 2016). The implications of this study are twofold. (1) It suggests that the pathogenesis of alpha-synucleinopathies in the brain may not entirely depend on the direct transport of alpha-synuclein from the ENS to the CNS but instead may develop in the brain as a result of SCFAs. (2) It supports the hypothesis that exposure to bacterial metabolites at peripheral sites in the body can induce pathology in the CNS. Further, the observation that no motor deficits were seen in germ-free (GF) mice upon exposure to heat-killed bacteria lends support for the theory that SCFAs are actively produced metabolites that are capable, and perhaps potentially necessary, for microglial activation and distant alpha-synuclein aggregation in the brain (Sampson et al., 2016). The way that SCFAs interact with alpha-synuclein in the gut to cause alpha-synuclein pathology in the brain is an ongoing part of research. The observation that some patients with vagotomies go on to develop PD lends further support for the presence of alternative routes to vagal transport of alpha-synuclein from the ENS to the CNS (Svensson et al., 2015). These findings add to the growing body of evidence that the microbiota induces alpha-synuclein aggregation and that in some cases this interaction appears to be independent of the vagus nerve.

## The Microbiota’s Role in Host Health

The last decade has seen major breakthroughs in identifying biological mechanisms used by our immune system in recognizing and interacting with the diverse species of microorganisms that make up our microbiota. The communication between these microorganisms and our own cells is mediated by a large number of pathways including the vagus nerve, the bloodstream and the endocrine system. In the late 1990s, researchers discovered PRRs, as part of the innate immune system that identify microorganisms based on unique molecular structures (Thaiss et al., 2016b). Activation of these PRRs, such as the activation of Toll-like receptors (TLRs) and nucleotide-binding oligomerization (NOD)-like receptors (NLRs) by bacteria, viruses, and fungi lead to a host of responses by the immune system (Honda and Littman, 2016; Caputi and Giron, 2018; Wang et al., 2019). The fact that (1) the constant presence of bacteria does not continuously activate these receptors and (2) certain types of bacteria are capable of stimulating the receptors suggests that the microbiota is modulating our immune responses in a controlled manner.

Further evidence for this controlled modulation can be found in the way subsets of gut bacteria interact with enterochromaffin cells (ECs) to affect host serotonin (5-HT) synthesis in the colon. The observation that repopulating the microbiota with bacteria that fail to interact with enterochromaffin cells (ECs) has no effect on 5-HT synthesis suggests that this process is tightly controlled and dependent on the presence of particular gut bacteria (Yano et al., 2015). Even though it is widely recognized that the microbiota plays a critical role in maintaining human health, the extent that these commensal organisms exert their influence on our brains is only beginning to be realized. It is becoming increasingly clear that the cellular and molecular contributions of the microbiota have an important role in maintaining and regulating healthy neuronal function. In a recent study, mice with gamma-aminobutyric acid (GABA) producing *Bifidobacterium* strains in their guts exhibited markedly decreased visceral pain responses compared to mice with *Bifidobacterium* not capable of producing GABA (Pokusaeva et al., 2017), suggesting that bacterial metabolites produced by gut bacteria are capable of eliciting responses in the CNS of mammals. Enteric bacteria have been found to produce a wide number of molecules involved in neuro-communication such as SCFAs, 5-HT, histamine, catecholamines and glutamate (Mazzoli and Pessione, 2016; Endres and Schäfer, 2018; Kwon et al., 2019; Ratajczak et al., 2019). Together, these observations not only demonstrate that neurotransmitter production by the microbiota may be a means of microbiota-gut-brain communication but also that changes in the gut microbiota have the capacity to exert measurable influence over the CNS.

### How Dysbiosis Contributes to CNS Disorders

Operating in close relationship to our ENS and the environment of our gastrointestinal tract, the microbiota has proven to be integral in the development and maintenance of our innate immune system. This is intuitive when you think about the fact that everything we ingest, from the foods we eat to the toxins we are exposed to, pass along our gastrointestinal tract. Perhaps the most striking evidence for the role of the microbiota in this process comes from observing what happens to the host when the microbiota is altered. GF mice have been shown to exhibit developmental defects at the molecular, cellular and tissue level (Round and Mazmanian, 2009; Honda and Littman, 2016; Thaiss et al., 2016a). This immunological dysregulation has been suggested as the cause of several non-infectious diseases ranging from autoimmune conditions and allergies to cancer (Round and Mazmanian, 2009; Honda and Littman, 2016; Mazzoli and Pessione, 2016; Thaiss et al., 2016b; Lammert et al., 2018; Ratajczak et al., 2019). Given the central role that the microbiota plays in the development of healthy functioning immune systems, it is reasonable to speculate that disruptions in the microbiota have the potential to drive immune dysfunction.

Evidence for the highly sophisticated ways bacteria have co-evolved within hosts to maximize fitness is documented throughout all the kingdoms of life. From the presence of mitochondria in animals and chlorophyll in plants, evidence for endosymbiotic relationships between bacteria and their hosts dates back billions of years. An example of this can be seen in present day interactions between bacteria in the gastrointestinal system of mammals and host cells. Human-derived or dietary glutamate (Glu) for instance has been shown to affect the gut microbiota by stimulating the growth of certain bacteria at the expense of others (Mazzoli and Pessione, 2016). The *Lactobacillus* genus possesses LAB decarboxylase (an enzyme that provides them with enhanced metabolic energy) that gives them an edge up on other bacteria lacking this enzyme (Mazzoli and Pessione, 2016). Another way that bacteria increase their fitness is by the implementation of defenses that keep them from being destroyed. One of the mechanisms that bacteria have evolved as a defense is the endotoxin lipopolysaccharide (LPS). LPS is the main component of the outer membrane of gram-negative bacteria that functions as a slippery coating that makes it difficult for cells of the immune system to phagocytose and destroy them.

Several recent clinical and experimental studies have demonstrated the existence of altered gut microbiota and microbial metabolites in various CNS and neuropsychiatric disorders (Bedarf et al., 2017; Lammert et al., 2018; Sun and Shen, 2018). Psychiatric disorders such as schizophrenia and autism have historically been associated with congenital predispositions, but the reason for this association remains unclear. The microbiota may offer a possible explanation. Evidence supporting the microbiota’s role in these associations can be seen in changes observed in host cognition and behavior after fecal microbiota transplants (FMTs) (Stolzenberg et al., 2017; Lammert et al., 2018). In a recent study, researchers looked at the relative abundance of *Enterobacteriaceae* in patients with PD and found that (1) patients had higher levels of these bacteria relative to healthy controls, and (2) the abundance of this family was positively associated with severity of postural instability and gait difficulty (Scheperjans et al., 2015). Analysis of fecal samples from patients with PD reveal decreased proportions of certain species of bacteria like *Faecalibacterium prausnitzii* as well as decreased levels of the SCFAs acetate, propionate, and butyrate (Keshavarzian et al., 2015; Unger et al., 2016). As previously stated, SCFAs are generally associated with maintaining the blood-intestinal and blood-brain barriers and are one of a select few substances that can cross the blood-brain-barrier. Decreased levels of SCFAs, as is observed in the microbiota of patients with PD then, offers a plausible mechanism for increased gut-blood-brain permeability and secondary exposure to environmental and bacterial triggers thought to induce alpha-synuclein aggregation in the ENS (Figure 1). Of note, in the study performed by Sampson et al. (2016) the profile of the SCFAs that induced Parkinsonian like phenotypes in mice receiving the oral gavages was significantly altered compared to controls. This observation suggests that there may be SCFAs that are more beneficial than others and that these metabolites likely affect the blood barriers of the gut and brain on a continuum.

## Alpha-Synuclein in the Microbiota-Gut-Brain Axis: A Role in Innate Immunity

### Gut Microbiota and Alpha-Synuclein

Another way that the enteric microbiota has been implicated in local and systemic inflammatory processes is with bacteria derived proteins (Codolo et al., 2013; Thaiss et al., 2016a; Caputi and Giron, 2018). While the exact mechanisms of how these proteins interact with alpha-synuclein are unknown, the work of several recent studies suggests that the bacterial LPS may play a large role in the inflammatory process of neurodegenerative disease (Changyoun et al., 2016; Stolzenberg et al., 2017; Terada et al., 2018). In a recent study, mice who received intracerebral injection of alpha-synuclein and were exposed to LPS positive bacteria produced a distinct fibrillar form of alpha-synuclein compared to mice exposed to LPS negative bacteria (Changyoun et al., 2016). The observation that exposure to LPS causes alpha-synuclein to adopt a fibrillar form and the specific patterns of alpha-synucleinopathies seen in mice exposed to this form of alpha-synuclein suggests that bacterial exposure may be a driving force in alpha-synucleinopathies. LPS has also been shown to induce expression of chemokines (Leow-Dyke et al., 2012; Honda and Littman, 2016; Popichak et al., 2018; Lee et al., 2019). Recent studies looking at the mechanisms underlying this interaction have identified an important role of the activation of TLR-4 causing the release of chemokines CCL5/RANTES and CXCL1 as well as TNF-alpha and IL-6 (Leow-Dyke et al., 2012; Perez-Pardo et al., 2018b; Figure 1).

### Alpha-Synuclein, Inflammation, and Immunity

In addition to playing a role in neurotransmitter release, alpha-synuclein has also been shown to have chemoattractant activity. Similar to the way that cytokines such as IL-18, IL-22, and IL-23 function as important messengers for initiating antimicrobial responses in the epithelium of the intestine (Thaiss et al., 2016b), it has been proposed that aggregated alpha-synuclein may function as a messenger to alert the immune cells in the CNS to the presence of certain pathogens. Several recent studies have observed aggregated alpha-synuclein causing activation and migration of neutrophils, microglia and dendritic cells in the CNS (Sampson et al., 2016; Sun and Shen, 2018). Interestingly, recent studies have also observed that microglia cell development and activation appears to be under the control of the gut microbiota (Erny et al., 2015; Sun and Shen, 2018). The microglia in the brains of mice under GF conditions not only display a very different morphology than the microglia in the brains of mice with intact microbiota but they also exhibit defects in maturation and function (Thaiss et al., 2016b). Similar observations have been seen in studies looking at the impact changes in the microbiota have on immune system responses upon exposure to viruses (Stolzenberg et al., 2017; Vitbarek et al., 2018). A recent study looking at what happens when the microbiota is deleted found that GF mice exposed to the lymphocytic choriomeningitis virus (LCMV) had a greatly disturbed innate immune response compared to mice exposed to the virus with intact microbiota (Erny et al., 2015). Not only was microglia density decreased in the GF mice, but PCR analysis revealed significant changes in important innate immune pathways such as c-Jun, c-Fos, FosB, cyclin B2 and proinflammatory molecules like TNF-alpha and IL-1beta in the GF mice (Erny et al., 2015). One explanation for this observation is that by controlling immune function at the level of microglia, the gut microbiota prepares the brain to mount targeted immune responses against harmful pathogens (Erny et al., 2015). Given that aggregated alpha-synuclein is a chemoattract to microglia, it is conceivable that aggregated alpha-synuclein might function as one of the messenger molecules directing these responses.

### Gastrointestinal Inflammation and Parkinson’s Disease

A recent study done by Stolzenberg et al. (2017) looked at endoscopic biopsies from children with documented gastric and duodenal inflammation as well as intestinal allograft recipients shown to have contracted norovirus after transplant. They found that (1) alpha-synuclein was detected in the duodenum of the pediatric patients and positively correlated to the degree of inflammation of the intestinal wall and (2) *de novo* alpha-synuclein was detected in the duodenum of transplant patients during norovirus infection that remained elevated for the duration of infection (Stolzenberg et al., 2017). The results of this study, in conjunction with the strong association seen between PD and the presence of alpha-synuclein in the submucosa of the sigmoid colon (Visanji et al., 2017) supports gastrointestinal inflammation and infection as potential factors in the pathogenesis of PD. The discovery that alpha-synuclein is expressed during acute and chronic gastrointestinal infection (Stolzenberg et al., 2017) along with alpha-synuclein being repeatedly shown to directly activate immune cells (Erny et al., 2015; Sampson et al., 2016; Stolzenberg et al., 2017; Sun and Shen, 2018), not only provides strong support for the theory that alpha-synuclein is involved in activating inflammation in the gut but it also offers a plausible explanation for how the presence of alpha-synuclein in the gut may contribute to PD. In another study looking at the way microbiota-immune system interactions contribute to the development of behavioral abnormalities, researchers found that female mice exposed to a viral mimetic polyinosinic-polycytidylic acid (PolyI:C) before conception had altered prenatal microbiota and their offspring exhibited behavior consistent with neurodevelopment disease (Lammert et al., 2018). Using a recombinant AAV as a vector, Ulusoy et al. (2013) found that injection of human alpha-synuclein into the vagus nerve of mice caused deposition of alpha-synuclein in the brain in a concentration, time and spatially dependent manner. In both of these studies, significant alterations in the brains of the rodents were observed after the introduction of viral mimetic particles (Lammert et al., 2018) or direct injection of virus particles (Ulusoy et al., 2013). While it is more likely that the presence of alpha-synuclein was driving the brain pathology in the study conducted by Ulusoy et al. (2013), it cannot be ruled out that viral particles may contribute to neurological pathology. With recent studies suggesting that bacteriophages outnumber the bacterial component of the microbiota 10:1 and appear to directly affect not just the populations of bacteria and fungi in the gut but the integrity of the blood-gut barrier as well (Dalmasso et al., 2014; Tetz and Tetz, 2016, 2018; Górski et al., 2017; Tetz et al., 2017) it will be important for future studies to more closely examine the potential role of viruses in PD.

## Future Directions in the Detection and Treatment of Parkinson’s Disease

### Biomarkers

Whether an intervention gets incorporated into a treatment regime for a patient is largely determined by the effect it has on the progression of the disease. The way scientists and physicians determine this is with the use of biomarkers, biological markers that are objectively measured and evaluated for the presence or absence of disease. Biomarkers are also useful in predicting a patient’s response to treatment and disease progression. When it comes to diseases involving the brain, particularly mental health and neurodegenerative disorders, finding biomarkers becomes more difficult. Surrounded by three layers of protective coverings and encased on all sides in a vault of bone, our CNS has evolved to be highly protected from insult. These defenses make it difficult to “see” how the brain is affected under different conditions, posing a unique set of challenges for scientists trying to develop biomarkers to assess CNS function. Measuring the amount of free water in the posterior substantia nigra on magnetic resonance imaging (MRIs) of patients with PD has been shown to be a strong indicator for the progression of PD by providing an indirect measure of dopaminergic degeneration and the subsequent clinical changes that follow (Ofori et al., 2015). Moreover, the short-term increase in free water appears to be particularly indicative of long term disease progression with baseline levels of free water strongly predicting the rate of change in free water over 4 years (Burciu et al., 2017). Research conducted by Burciu and others measuring changes in markers like free water levels on MRI scans or alpha-synuclein sampling on lumbar punctures (Hall et al., 2018) offer ways to show brain degeneration progression in patients with PD. But, they also have significant drawbacks. First, these procedures are expensive and may lead to a significant financial burden on the patient, particularly if performed on a routine basis. Second, by the time patients exhibit neurological symptoms, significant neurodegeneration has already occurred. Finally, sampling cerebrospinal fluid via lumbar punctures involves an invasive and painful procedure the patient is unlikely to want to repeat. Studies demonstrating that PD pathology is not confined to the CNS and that it may, in fact, manifest first in the periphery offer exciting opportunities for the development of non-invasive biomarkers. Future research should therefore focus attention on the development of biomarkers that assess changes in peripheral involvement.

Studies like those conducted by Keshavarzian et al. (2015), Scheperjans et al. (2015), Caputi and Giron (2018) demonstrate that patients with PD have a different composition of their microbiota and that certain species of bacteria correlate to the severity of neuromotor symptoms. These observations suggest that bacteria taxonomy could be a potential biomarker in identifying possible risk factors for the development of PD. A recent study looking at routine colonic biopsies collected for colorectal screens found that patients who had confirmed clinical diagnoses of PD stained positive for alpha-synuclein years before any onset of PD symptoms, findings that were absent in healthy controls (Shannon et al., 2012). This is significant not just because it lends further support for the hypothesis that PD may first appear in the gut, but perhaps more importantly, it suggests a novel use of colonic biopsies as a biomarker for early detection of PD (Shannon et al., 2012). When done correctly, use of sigmoid colon biopsies to detect the presence of aggregated alpha-synuclein may have a 100% sensitivity and specificity for patients later developing pathologically advanced PD compared to controls (Visanji et al., 2017). Research addressing what to do with this knowledge and how detection of alpha-synuclein in the periphery may 1 day lead to changes in therapy has yet to be undertaken. However, with the increasing use of colonoscopies and fecal samples being collected to detect colon cancer, it seems plausible that the same techniques may 1 day be used to predict a person’s risk of developing PD.

Potential targets for the development of future biomarkers to measure the progression of pathology in PD are summarized in Table 1.

**Table 1:** Potential targets for the development of future biomarkers in measuring the progression of pathology in Parkinson’s disease (PD).

Biomarker	Association	Proposed contribution to pathogenesis	Source of biomarker	References
Fecal levels of acetate, propionate and butyrate (SCFAs)	Reduced in patients with PD	–Decreased SCFAs levels leads to decreased levels of sodium-butyrate, a histone deacetylase inhibitor that protects dopaminergic neurons – Decreased SCFAs result in decreased motility of gut, driving non-motor gastrointestinal dysregulation seen in PD	Fecal sample	Unger et al., 2016
*Faecalibacterium prausnitzii*	Reduced in patients with PD	– Low levels associated with decreased levels of butyrate – Low levels associated with loss of IL-10 secreting T-regulatory cells (Tregs)	Fecal sample	Sarrabayrouse et al., 2014; Keshavarzian et al., 2015; Honda and Littman, 2016; Unger et al., 2016
*Prevotellaceae*	Reduced in patients with PD	– Low levels decrease mucin synthesis, increasing gut permeability leading to environmental exposure – Low levels correspond to increased alpha-synuclein expression in colon – Low levels lead to loss of neurodegeneration restriction by impairing ghrelin secretion pathway	Fecal sample	Scheperjans et al., 2015; Unger et al., 2016; Bedarf et al., 2017
*Enterococcaceae*	Reduced in patients with PD	Unknown	Fecal sample	Unger et al., 2016
*Enterobacteriaceae*	Elevated in patients with PD	– Increases permeability of intestinal epithelium exposing ENS to environmental toxins – Positive association with postural and gait instability in PD patient population	Fecal sample	Keshavarzian et al., 2015; Scheperjans et al., 2015; Unger et al., 2016
*Bifidobacterium*	Elevated in patients with PD	unknown	Fecal sample	Keshavarzian et al., 2015; Scheperjans et al., 2015; Unger et al., 2016; Hopfner et al., 2017
Alpha-synuclein in sigmoid mucosal neurons	Present 2–5 years before onset of PD motor symptoms	– Aggregations in sigmoid colon observed in patients with PD prior to diagnosis	Sigmoid mucosal biopsies	Shannon et al., 2012; Visanji et al., 2017
*Lachnospiraceae*	Reduced in patients with PD	– Low levels associated with decreased levels of butyrate	Fecal sample	Keshavarzian et al., 2015; Bedarf et al., 2017


### Future Therapies in the Management of Parkinson’s Disease

Currently, there are no therapies that are effective at stopping or even slowing the progression of PD. Current treatment can only offer patients brief respites from symptoms and is successful at decreasing the severity of tremors and dyskinesia in only some patients. Inevitably these interventions fail either because of tolerance to pharmacologic agents like Levodopa or secondary to progressive degeneration.

In a systematic review of the prevalence of depression in PD, depression was reported in up to half of all patients suffering from PD (Reijnders et al., 2008). While standard treatment of depression with antidepressants such as selective serotonin reuptake inhibitors (SSRI) can have a tremendous effect on improving mood in most patients with depression, SSRI use in patients with PD is controversial for two reasons. First, they have been shown to aggravate the motor symptoms of the disease (Bharucha and Sethi, 1996). Second, they have potential to negatively interact with the drug Selegiline, a monoamine-oxidase type B (MAO-B) inhibitor sometimes used in the treatment of PD (Richard et al., 1997). Finding an alternative therapy to standard treatment of depression for patients with PD is therefore critically important. One alternative for treating depression may be to target the source of serotonin production itself. An estimated 90% of all serotonin produced in our bodies comes from the enterochromaffin cells of the gut (Caputi and Giron, 2018). Studies done by Yano et al. (2015) demonstrate direct evidence that serum levels of serotonin can be raised by introduction of certain spore-forming bacteria. The use of neuromodulating bacteria, so called psychobiotics (Mazzoli and Pessione, 2016), for the treatment of mood disorders represents an exciting potential therapy for the millions of patients with PD suffering from depression and anxiety.

The changes seen in the immune pathways that accompany PD, particularly those involved in the pro-inflammatory cascade, represent another potential target in the development of future treatments. A retrospective cohort study found that patients with a diagnosis of irritable bowel disease that were exposed to anti-TNF therapy resulted in a 78% reduction in the incidence rate of PD compared to patients that did not receive anti-TNF therapy (Peter et al., 2018). The use of immune modulating therapies in the brain may represent another potential therapy. Postmortem brain examination from patients with PD revealed that alpha-synuclein pathology was accompanied by HLA-DR+ (a MHC class II cell surface receptor) reactive microgliosis, increased pro-inflammatory cytokine expression and infiltration of peripheral lymphocytes (Harms et al., 2013; Ferreira and Romero-Ramos, 2018; Figure 1). Targeting this process with immune modulating therapies that block pro-inflammatory cytokines like TNF, IL-1beta, and interferon gamma may be effective at preventing microgliosis and immune activation in the brain. Lymphocyte-activation gene 3 (LAG3) has recently been identified as a receptor of misfolded alpha-synuclein and appears to play a role in propagation of alpha-synuclein pathology as transmission from one neuron to the next is initiated by binding of LAG 3 (Mao et al., 2016). If LAG3 is indeed central to the spread of pathological alpha-synuclein, development of an antibody against LAG3 may represent another treatment in slowing the progression of PD.

The involvement of TLRs in the activation of microglia has been another active area of research. Researchers looking at the influence of alpha-synuclein oligomers on TLR2 found that blocking TLR2 decreased neuronal inflammation in mice by reducing glial activation, neuronal loss, and cytokine gene expression (Kim et al., 2015). Targeting TLR2 in future therapies may decrease neuroinflammation by (1) dampening the immune response by decreasing microgliosis and (2) disrupting alpha-synuclein deposition (Kim et al., 2015; Caputi and Giron, 2018). Targeting the JAK-STAT pathway, an important cellular signaling pathway involved in immunity and cell division, may represent yet another way to reduce neuroinflammation. In rat models, suppression of the JAK-STAT pathway has been shown to inhibit alpha-synuclein induced microglia activation, macrophage activation and CD4+ T-cell infiltration in the CNS (Qin et al., 2016). Designing treatments around suppressing this pathway with JAK-STAT inhibitors may be another option for patients with PD disease (Qin et al., 2016; Ferreira and Romero-Ramos, 2018). Collectively these findings not only strengthen the hypothesis that inflammation plays a major role in PD, but they also support the growing consensus that PD is a systemic inflammatory disease and that exploring immune modulating therapies is critical for the prevention of PD.

With emerging studies showing an association between PD and the abundance of certain gut microbiota (Keshavarzian et al., 2015; Scheperjans et al., 2015; Sampson et al., 2016; Unger et al., 2016) cultivating healthy gut microbiota with bacteria like *Prevotellaceae* (a family found to be depleted in the microbiota of patients Autism-Spectrum Disorder and PD believed to maintain healthy gut and blood-brain barriers) (Keshavarzian et al., 2015) and closely monitoring levels of *Enterobacteriaceae* (a family found to be disproportionally increased in patients with PD) (Scheperjans et al., 2015; Unger et al., 2016) may represent novel ways of promoting brain health. Dietary changes have the potential of benefiting patient health by modulating the abundance of different types of bacteria that make up the gut microbiota, decreasing inflammation and reducing barrier permeability. Dietary interventions therefore represent another approach in the treatment and maybe even prevention of neurodegenerative and CNS disorders. A recent study looking at the effects of dietary changes on mice exposed to the toxin rotenone found that dietary supplement with uridine and docosahexaenoic acid (DHA) corresponded to decreased motor and GI abnormalities compared to mice exposed to rotenone without dietary supplement (Perez-Pardo et al., 2018a). Ma et al. (2018) found that after being on a ketogenic diet for 16 weeks, mice showed significant increases in cerebral blood flow, an increase in relative abundance of putatively beneficial bacteria *Akkermansia muciniphila* and *Lactobacillus*, and a stronger blood-brain-barrier compared to mice on a standard diet. Future studies looking at the mechanisms by which diet influences the microbiota-gut-brain axis are needed to create more targeted therapies in the prevention and treatment of PD.

In a recent study looking at the microbiota of patients with PD, healthy controls and patients with idiopathic rapid eye movement sleep behavior disorder (iRBD) found significant overlap in the composition of microbial taxa between patients with PD and patients with iRBD compared to controls (Heintz-Buschart et al., 2018). The observation that patients with iRBD, a disorder that strongly correlates with future PD diagnosis, have similar microbiota composition as patients with PD supports FMTs as a potential future therapy. Given that iRBD usually develops years before patients are diagnosed with PD, repopulating patients with iRBD with protective species via FMTs may be an effective therapy in preventing PD. With the recent success that FMTs have had in correcting dysbiosis after *Clostridium difficile* infections and the emerging evidence that dysbiosis exists in patients with PD, it is plausible that FMTs may 1 day be used in treating PD.

### Limitations of Current Research

Arguably multiple levels of complexity exist to hinder the detailed understanding of the relationship between the microbiota and PD. One obstacle is that the microbiota-gut-brain axis communicates via many different pathways such as neuronal, immune and endocrine. A second obstacle is alpha-synuclein pathogenic species have been shown to develop under a myriad of conditions and in response to a number of different stimuli which makes it difficult to identify causative agents. Additionally, the current models outlining ways by which pathogens initiate disease in alpha-synucleinopathies are mainly speculative since not all signaling molecules and receptors have been fully identified. Moreover, it is also unclear why the microbiota of patients with PD is altered. These challenges make it difficult to ascribe clear conditions that render an individual more at risk for developing PD than any other person. With recent studies providing evidence that environmental triggers like the presence of overabundant strains of LPS producing bacteria or certain viral infections appear to be associated with pathological alpha-synuclein deposition in the gut and brain, there is reason to seriously consider the idea that peripheral changes may be, in part, driving PD and other alpha-synucleinopathies. Further understanding of alpha-synuclein and its relationship to the microbiota-gut-brain axis could reveal new therapeutic targets for the prevention and treatment of PD.

## Conclusion

The understanding of the mechanical and chemical mechanisms by which changes in the microbiota contribute to neurodegenerative disorders like PD is in its infancy. In the last decade, it has become increasingly clear that the microbiota is capable of modulating a series of responses in humans from simple changes in appetite to more complex behaviors like emotion and memory. The notion that changes in the microbiota have direct effects on brain health has been well established. With developments like the identification of TLRs and mechanisms by which bacterial metabolites interact with microglia and other neural cells, our comprehension and appreciation for the extremely sophisticated communication between the microbiota and our cells is growing. With the estimated number of patients diagnosed with PD to more than double in the next 20 years (Dorsey and Bloem, 2018) elucidating the details of the microbiota’s role in the pathogenesis of this devastating disease is particularly relevant. With recent studies providing evidence that PD may begin, or at the very least co-manifest itself in the gut, it is imperative that future research look at the environment of the GI tract, particularly how changes in the environment and normal flora contribute to the disease.

## Author Contributions

EF, SM, and HM conceptualization and review and editing. EF original draft preparation. HM funding acquisition.

## Conflict of Interest Statement

The authors declare that the research was conducted in the absence of any commercial or financial relationships that could be construed as a potential conflict of interest.
